# Comment on Viderman et al. Remote Monitoring of Chronic Critically Ill Patients after Hospital Discharge: A Systematic Review. *J. Clin. Med.* 2022, *11*, 1010

**DOI:** 10.3390/jcm12206433

**Published:** 2023-10-10

**Authors:** Parisa Farahani, Lana Wahid

**Affiliations:** Division of General Internal Medicine, Department of Medicine, Duke University Medical Center, Durham, NC 27710, USA; lana.wahid@duke.edu

Viderman et al. undertook this systematic review with the intent of providing evidence on the potential application of remote monitoring in chronic critically ill patients post-hospital discharge [1]. However, a critical examination of their systematic review casts doubts on the evidence they provide to support their claim. Several issues warrant consideration:

Firstly, the research protocol submitted to PROSPERO is about the utilization of devices in the context of brain injuries. Curiously, the authors broaden their research question to encompass critically ill patients. This shift becomes more questionable given that the database search was conducted on 15 May 2021, while the protocol submission followed on 18 June 2021 without apparent alignment with the search strategy. Notably, the keywords and terms for these two topics are quite different, so it is unclear how conclusions can be drawn about remote monitoring of critically ill patients while their original focus was on brain injuries.

On the other hand, while the focus of this study centers on the utilization of remote monitoring devices post-discharge; the included papers mainly address deployment of these devices in inpatient settings and their validation in healthy individuals. A notable gap in this systematic review is the lack of papers addressing the utilization of remote monitoring in patients after their hospital discharge. 

While this review does highlight significant aspects of the remote monitoring and biometric devices era, it is constrained by the questionable validity of its literature search. As a result, it leans more toward a literature or scoping review that addresses remote patient monitoring as a broader health-related concern, rather than a meticulously conducted systematic review. Readers should be mindful of these limitations when interpreting these findings and applying them to their future research.

Also, further systematic reviews with more rigorous search strategies and stricter inclusion and exclusion criteria are imperative to address this crucial healthcare issue.
